# The effects of a structured education program on glycaemic control in individuals with type 1 diabetes

**DOI:** 10.1186/1758-5996-7-S1-A53

**Published:** 2015-11-11

**Authors:** Ana Paula Franco Pacheco, Simone Van de Sande Lee, Cristina S Schreiber Oliveira, Julia Michels, Rita Bruno Sandoval, Jefferson Luiz Brum Marques

**Affiliations:** 1 UFSC, Florianópolis, Brazil

## Background

According to the IDF the number of cases worldwide reached 387 million in 2014, while Brazil occupies the 4th place in the overall ranking with over 13 million cases. These data refer to both Type 2 Diabetes (T2D) and T1D. Although T1D affects the minority of patients, it is responsible for many of the serious complications. T1D, usually diagnosed in youth, requires continuous treatment and control that demand care such as diet, medication and lifestyle changes. This project addresses the importance of a Structured Education Program (SEP) for self-care of individuals with Type 1 Diabetes Mellitus (T1D).

## Objective

To observe whether the SEP has any positive effect in the treatment of T1D by providing individuals with information about their health-disease situation, leading to an increased awareness, providing skills to become able to manage their condition effectively.

## Materials and methods

Forty-seven T1D individuals were followed for 20 months. Values of HbA1c were analyzed and compared. Questionnaires, workshops, individual care, 24-hour support, and semi-structured interview were applied.

## Results

**Figure 1:**
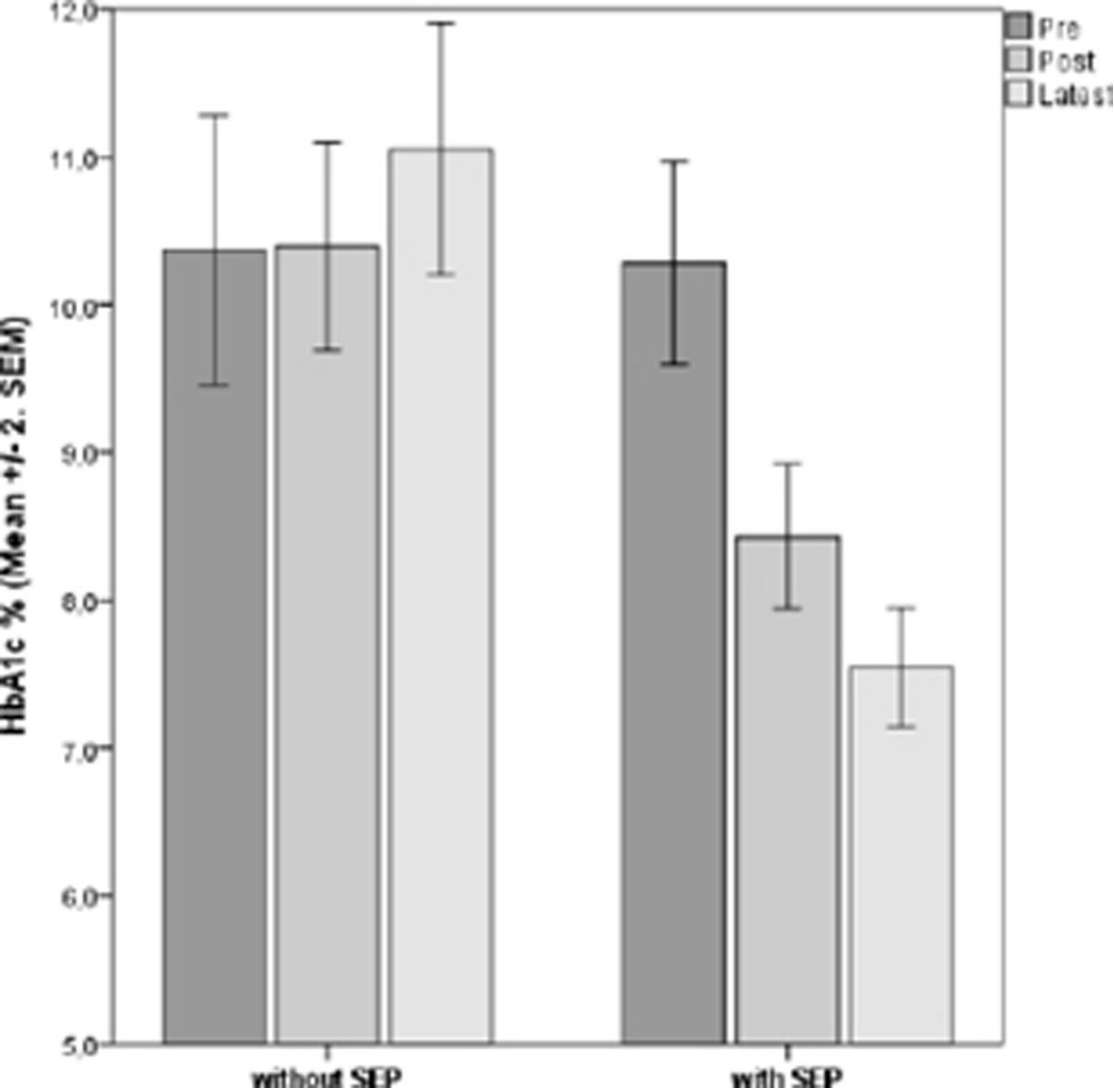
Means±SEM (%) related HbA1c values of 47 Individuals (Pre, Post and Latest) and (with and without SEP).

**Figure 2:**
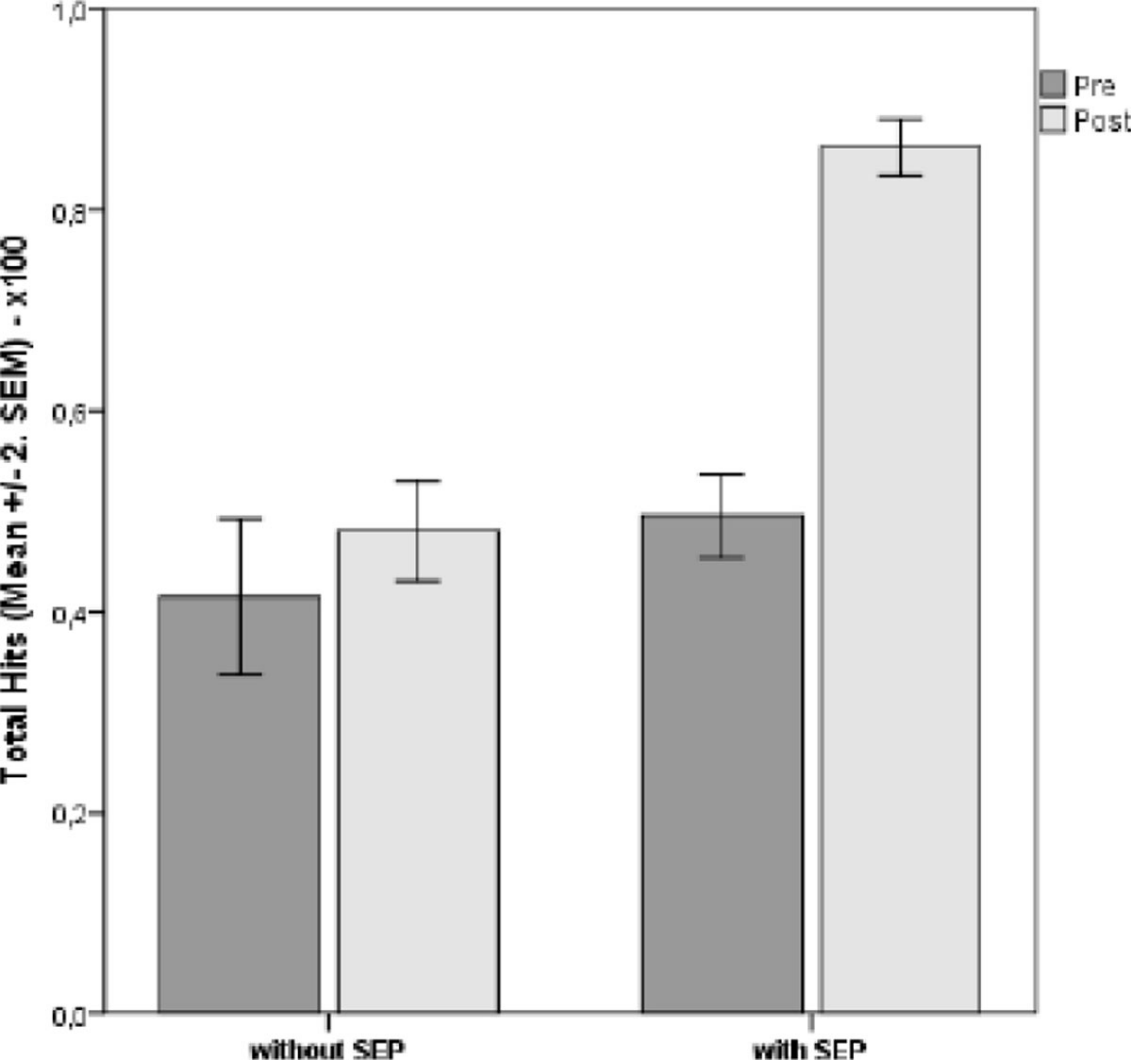
Means±SEM (%) related to items of category Total Hits Questionnaire pre and post SEP. Comparison between individuals with and without SEP.

Mean HbA1c decreased 1.9% (21 mmol/mol) in one year and a further reduction of 0,9% (10 mmol/mol) was observed after 8 months (p<0.001).(Figure 1) Evaluation of overall knowledge showed an increase of 37% when comparing the pre- and post-SEP (p<0.005). (Figure 2) Feedback showed 87% improvement of self-management; 81% were more satisfied with current quality of life; 94% acknowledged diet flexibility; 91% were highly motivated to continue treatment appropriately (p<0.005).

## Conclusion

Relevant clinical and psychological improvements were demonstrated. The average decrease in HbA1c, the overall knowledge and confidence increase and improved quality of life confirms the importance, necessity and positive outcome of a SEP in diabetes.

